# Low Virologic Failure and Drug Resistance among HIV-Infected Patients Receiving Hospital-Based ART While Care and Outreach through Community in Guangxi, China

**DOI:** 10.3389/fpubh.2015.00244

**Published:** 2015-10-27

**Authors:** Shujia Liang, Zhiyong Shen, Jing Yan, Fuxiong Liang, Zhenzhu Tang, Wei Liu, Wei Kan, Lingjie Liao, Xuebing Leng, Yuhua Ruan, Hui Xing, Yiming Shao

**Affiliations:** ^1^Guangxi Center for Disease Control and Prevention, Nanning, China; ^2^State Key Laboratory of Infectious Disease Prevention and Control, National Center for AIDS/STD Control and Prevention, Beijing, China; ^3^State Key Laboratory for Infectious Disease Prevention and Control, Chinese Center for Disease Control and Prevention, Beijing, China; ^4^State Key Laboratory for Infectious Disease Prevention and Control, Collaborative Innovation Center for Diagnosis and Treatment of Infectious Diseases, Beijing, China

**Keywords:** HIV, antiretroviral treatment, virologic suppression, drug resistance, China

## Abstract

**Objectives:**

To investigate human immunodeficiency virus (HIV) virologic suppression and drug resistance among HIV-infected patients receiving first-line antiretroviral treatment (ART) in hospitals while community care and outreach through local health workers in Guangxi, China.

**Design:**

This was a series of cross-sectional surveys from 2004 to 2012 in Guangxi, supported by the Chinese National HIVDR Surveillance and Monitoring Network Working Group.

**Settings:**

Guangxi, China.

**Methods:**

Demographic, ART, and laboratory data (CD4^+^ cell count, viral load, and drug resistance) were analyzed. Factors associated with virologic suppression were identified by logistic regression analysis.

**Results:**

A total of 780 patients were included in this study. The median treatment duration was 20.6 months (IQR 6.6–35.9). Of 780 study participants, 95.4% of patients (744/780) had HIV virologic suppression. Among these, of the 143 patients who were infected through drug injection, only 10 (7.0%) experienced virologic failure, and the overall prevalence of HIV drug resistance was 2.8% (22/789). Factors associated with virologic suppression in the final multivariate models included self-reported missing doses in the past month (compared to not missing doses in the past month, AOR = 0.2, 95% CI: 0.1–0.6) and initial ART regimen without 3TC (compared to initial ART regimen with 3TC, AOR = 0.2, 95% CI: 0.1–0.4). Moreover, the trend chi-square test showed that the proportion of virologic suppression increased over time from 2004 to 2012 (*P* = 0.002).

**Conclusion:**

This study first demonstrated that HIV patients infected through various transmission routes can achieve an excellent treatment outcome in hospitals at or above the county level for free first-line ART in Guangxi. It is an important of ART education and adherence to intervention for achieving better treatment outcomes.

## Introduction

Highly active antiretroviral therapy (HAART) has led to a dramatic increase in sustained suppression of human immunodeficiency virus (HIV) replication. Since HAART was developed in the early 1990s, there has been a rapid improvement in prognosis of patients infected with HIV and reduction in AIDS-related mortality (1, 2). In resource-rich countries, because of the use of optimal regimens and a complete monitoring system for virologic failure and drug resistance, levels of drug resistance decreased over time (3, 4). As a result of the scale-up of the “3 by 5” project in low-income and middle-income countries, an increasing number of patients received antiretroviral treatment (ART) from 2003. However, the emergence of HIV drug resistance (HIVDR) was a big challenge to the rapid development of HAART (5, 6).

The China’s National Free Antiretroviral Treatment Program (NFATP) began in 2002 and rapidly scaled-up. In 2008, China’s NFATP changed the threshold of initiating free ART access to more patients (7). By the end of 2013, among the 436,817 people living with HIV in China, the total number receiving free ART had increased to 278,080 (8). In China, ART has significantly reduced mortality among HIV patients (9–11) and HIV transmission among serodiscordant couples in the Chinese national observational cohort study (12). However, meta-analysis study results showed that the pooled prevalence of HIVDR was 11.1% during 0–12 months and increased to 22.92% at 61–72 months in Chinese HIV-Infected Patients Receiving First-Line HAART (13). Guangxi Zhuang Autonomous Region located on the southern coast of the China, and neighboring Vietnam in the South and Yunnan Province in the west, is the major drug trafficking route linking Guangxi with Yunnan and Vietnam. The reported cases of HIV/AIDS in Guangxi accounted for 10% of the reported numbers in China. The first HIV infection among local IDUs was observed in 1996 in Guangxi, and HIV infection through drug injection accounted for 69% of the cumulative reported cases of HIV in Guangxi in 2003 (14). However, in 2013, 93% of reported HIV cases in Guangxi were infected through heterosexual intercourse (15). Guangxi Zhuang Autonomous Region has the highest number of reported HIV cases in China (15). Because of the capacity in county hospital-based care and management for free ART in Guangxi, the aim of this study was to evaluate virologic failure and drug resistance and associated factors among HIV-infected patients receiving first-line ART.

## Materials and Methods

### Study Design and Study Participants

We conducted a series of cross-sectional surveys of HIVDR in adult patients at regional representative ART clinics in Guangxi from 2004 to 2012. This protocol of Chinese National HIVDR Surveillance was adopted from the WHO recommended cross-sectional survey on acquired HIVDR in adult patients receiving ART, which has been described elsewhere (16–19). First-line ART regimens before 2008 consisted of [azidothymidine (AZT) or stavudine (D4T)] + [didanosine (DDI) or lamivudine (3TC)] + [nevirapine (NVP) or efavirenz (EFV)]. The second edition of the National Free ART Guideline in 2008 was revised to consist of [tenofovir (TDF) or azidothymidine (AZT)] + lamivudine (3TC) + [efavirenz (EFV) or nevirapine (NVP)] (7). The NFATP criterion for treatment was (1) CD4 cell count <200/mm^3^ before 2008, and <350/mm^3^ since 2008, (2) total lymphocyte count <1200/mm^3^ before 2008, and/or (3) WHO stage III or IV disease (7, 20). The study eligibility criteria were HIV patients, 18 years or older, having received first-line ART, willingness and consent to participate in the study. Patients who received second-line ART were excluded.

### Data Collection

The data were collected with an interviewer-administered questionnaire. Each participant provided informed consent before participation in the study. Each subject was assigned a confidential identification number to label questionnaires and blood specimens. The questionnaire was administered by trained local staff in a private room. The questionnaire information included demographic data, HAART treatment, and self-reported adherence measures. Demographic variables included sex, ethnicity, education level, occupation, marital status, and HIV transmission route. HAART treatment variables included initial regimen, the duration of HAART, and end of HAART regimen. Self-reported adherence variables included missed ART doses in the past month. The institutional review board (IRB) of the National Center on HIV/STD Control and Prevention (NCAIDS), China CDC approved this study.

### Laboratory Analysis

Blood specimens were provided by all subjects to determine CD4^+^ T-lymphocyte count (CD4 count), HIV viral load (VL), and HIVDR mutations. The CD4 count was determined by flow cytometry within 24 h after collection in the provincial CDC. The Plasma HIV RNA was quantified with real-time NASBA (NucliSense Easy Q, bioMerieux, France) or COBAS (Roche Applied Biosystems, Germany) according to the manufacturer recommendations. In samples with a viral load ≥1000 copies/ml, HIVDR was determined by using an in-house polymerase chain reaction (PCR) (16, 21). The Stanford HIV Drug Resistance Database (http://hivdb.stanford.edu/) was used to analyze drug resistance mutation and viral subtype determination. Low-, intermediate-, and high-level drug resistance were defined as HIVDR (22, 23).

### Data Analysis

Questionnaire and laboratory data were double entered and compared with Epidata 3.1 (The Epidata Association Odense, Denmark), and then were analyzed by Statistical Analysis System (SAS 9.2, SAS Institute Inc., Cary, NC, USA). Demographic variables were described with descriptive statistics. Factors associated with virologic failure (viral load ≥1000 copies/ml) were identified by univariate and multivariate logistic regression models. Variables with *P* < 0.05 were retained in multivariate logistic regression models using stepwise selection and results were presented with adjusted odds ratio (AOR) and 95% CIs. Hypothesis testing was two-sided with α = 0.05.

## Results

### Demographic Characteristics

Seven hundred eighty patients were included in this study. Among these, 221 patients were investigated in 2004–2006, 114 patients were investigated in 2006–2007, and then 291 patients were surveyed in 2007–2009, whereas 154 patients were included in 2011–2012. The demographic characteristics of the subjects are shown in Table 1. Among the patients, the mean age was 39.2 ± 12.2 years, 61.9% were male, 87.1% belonged to the Han ethnic group, 21.9% had an education level of high school or higher, 76.9% were primarily farmers, and 23.2% were married. Reported transmission routes were 75.3% sexual transmission, 18.3% injection drug use, 1.4% blood transmission, and 5.0% other.

**Table 1:** **Characteristics of HIV patients receiving first-line ART in Guangxi**.

Characteristics	Number	Percentage
Total	780	
Age (years): mean ± SD	39.2 ± 12.2	
Sex		
Male	483	61.9
Female	297	38.1
Ethnicity		
Han	679	87.1
Minorities	101	12.9
Education		
Illiterate	33	4.2
Primary school	236	30.3
Junior high school	340	43.6
High school or more	171	21.9
Occupation		
Others	180	23.1
Farmer	600	76.9
Married		
Yes	181	23.2
No	599	76.8
HIV transmission route		
Sexual intercourse	587	75.3
Drug injection	143	18.3
Blood transfusion	11	1.4
Others	39	5.0
CD4 count before ART (cells/mm^3^)		
≥350	10	1.3
349–200	116	14.9
199–100	142	18.2
<100	512	65.6
Missed doses in the past month		
No	757	97.9
Yes	23	2.1
Duration of ART (months): median, IQR	20.6	6.6–35.9
Initial ART regimen		
AZT + 3TC + EFV	95	12.2
AZT + 3TC + NVP	250	32.1
D4T + 3TC + EFV	40	5.1
D4T + 3TC + NVP	271	34.7
DDI-based regimens	124	15.9

### HAART Regimens and Virologic Profiles

Initial HAART regimens used were AZT + 3TC + EFV (12.2%), AZT + 3TC + NVP (32.1%), D4T + 3TC + EFV (5.1%), D4T + 3TC + NVP (34.7%), and DDI-based regimens (15.9%). The median of the duration of ART was 20.6 months. At the time of the survey, 36 (4.6%) patients had a viral load ≥1000 copies/ml. Among these subjects, 35 were successfully genotyped. CRF01_AE was the most common HIV viral subtype (31/35, 88.5%). Of these, 22 had detectable HIVDR mutations.

### HIV Drug Resistance Mutations

Among the 35 subjects successfully genotyped with viral load ≥1000 copies/ml, 22 patients were identified with HIVDR mutations, and the overall prevalence of HIVDR was 2.8% (22/789) among HIV-infected patients receiving first-line ART. Approximately 54.5% (12/22) of patients were resistant to non-nucleoside reverse transcriptase inhibitor (NNRTIs) drugs, 90.9% (20/22) of patients were resistant to nucleoside reverse transcriptase inhibitor (NRTIs) drugs, and 4.5% (1/22) of patients had drug resistance to protease inhibitors (PIs). In addition, 45.5% (10/22) of patients were identified as multi-drug resistance to NNRTIs and NRTIs. The most frequent NNRTIs mutations occurred at position 101 in the RT (reverse transcriptase) region, NRTIs mutations occurred at position 184 in the RT region, and PI mutations occurred at position 84 in the PR (protease) region (Table 2).

**Table 2:** **HIV drug resistance mutations among HIV patients receiving first-line ART in Guangxi**.

Antiretroviral drug	*N* (%)	HIV drug resistance mutations, *N* (%)
Total	22 (100.0)	
Non-nucleoside reverse transcriptase inhibitors (NNRTI, any)	12 (54.5)	K101E/EK/H/P, 4 (18.2)
Efavirenz (EFV)^a^	12 (54.5)	K103N/KN/S, 3 (13.6)
Nevirapine (NVP)^a^	12 (54.5)	V106L/I, 2 (9.1)
Delavirdine (DLV)	11 (50.0)	G190A, 2 (9.1)
Etravirine (ETV)	2 (9.1)	Y181C/I, 2 (9.1)
		E138EQ, 1 (4.5)
		Y188L, 1 (4.5)
Nucleoside reverse transcriptase inhibitors (NRTI, any)	20 (90.9)	
Emtricitabine (FTC)	18 (81.8)	M184V/MV, 8 (36.4)
Lamivudine (3TC)^a^	18 (81.8)	T69I/IN/N, 6 (27.3)
Abacavir (ABC)	15 (68.2)	D67DG/DN/G/N, 4 (18.2)
Didanosine (DDI)^a^	17 (77.3)	L74V, 2 (9.1)
Stavudine (D4T)^a^	14 (63.6)	M41I/LM, 2 (9.1)
Tenofovir (TDF)^a^	8 (36.4)	K65R, 1 (4.5)
Azidothymidine (AZT)^a^	12 (54.5)	V75T, 1 (4.5)
		L210M, 1 (4.5)
		F116Y, 1 (4.5)
		T215Y, 1 (4.5)
		K70KR, 1 (4.5)
		F215FIST, 1 (4.5)
Protease inhibitors (PI, any)	1 (4.5)	
Tipranavir (TPV)	1 (4.5)	I84IV, 1 (4.5)
Fosamprenavir (FPV)	1 (4.5)	
Lopinavir (LPV)^a^	0 (0.0)	
Nelfinavir (NFV)	1 (4.5)	
Atazanavir (ATV)	1 (4.5)	
Darunavir (DRV)	0 (0.0)	
Indinavir (IDV)	1 (4.5)	
Saquinavir (SQV)	1 (4.5)	
Multi-drug resistance to NNRTI and NRTI	10 (45.5)	

*^a^Provided through the National Free Antiretroviral Treatment Program (NFATP)*.

### Predictors for HIV Virologic Suppression

Of 780 study participants, 744 (95.4%) had HIV virologic suppression. Univariate logistic regression models were used to examine the factors associated with HIV virologic suppression (Table 3). Ethnicity, initial ART regimen, and missed doses in the past month were associated with virologic suppression. Those found to be statistically significant were included in the multivariate logistic regression model. Factors associated with virologic suppression in the final multivariate models include self-reported missing doses in the past month (compared to not missing doses in the past month, AOR = 0.2, 95% CI: 0.1–0.6) and initial ART regimen without 3TC (compared to initial ART regimen with 3TC, AOR = 0.2, 95% CI: 0.1–0.4). Moreover, the trend chi-square test showed that the proportion of virologic suppression increased over time from 2004 to 2012 (*P* = 0.002).

**Table 3:** **Factors associated with HIV viral suppression (viral load <1000 copies/ml) among HIV patients receiving first-line ART in Guangxi**.

Variable	Number	Viral suppression *N* (%)	Crude OR (95% CI)	*P*-value	Adjusted OR (95% CI)	*P*-value
	780	744 (95.4)				
Age (years)						
<40	486	458 (94.2)				
≥40	294	286 (97.3)	2.2 (0.9, 4.9)	0.06		
Sex						
Male	483	459 (95.0)				
Female	297	285 (96.0)	1.2 (0.6, 2.5)	0.55		
Ethnicity						
Han	679	652 (96.0)				
Minorities	101	92 (91.1)	0.4 (0.2, 0.9)	0.03		
Education						
High school or more	269	261 (97.0)				
Junior high school or less	511	483 (94.5)	0.5 (0.2, 1.8)	0.12		
Occupation						
Others	180	175 (97.2)				
Farmer	600	569 (94.8)	1.9 (0.7, 4.9)	0.19		
Married						
No	181	170 (93.9)				
Yes	599	574 (95.8)	1.5 (0.7, 3.1)	0.29		
CD4 cell counts before ART						
≥200	126	123 (97.6)				
<200	654	621 (96.3)	0.5 (0.1, 1.5)	0.20		
Initial ART regimen						
Regimens with 3TC	656	638 (97.3)				
Regimens without 3TC	124	106 (85.5)	0.2 (0.1, 0.3)	<0.0001	0.2 (0.1, 0.4)	<0.0001
HIV transmission route						
Drug injection	143	133 (93.0)				
Others	637	611 (96.0)	1.8 (0.8, 3.8)	0.14		
Missed doses in the past month						
No	757	726 (95.9)				
Yes	23	18 (78.3)	0.2 (0.1, 0.4)	<0.001	0.2 (0.1, 0.6)	0.003
Time of starting ART (years)						
Before 2006	182	165 (90.7)				
2006–2008	339	327 (96.5)	2.8 (1.3, 6.0)	0.008		
After 2008	259	252 (97.3)	3.7 (1.5, 9.1)	0.004		

## Discussion

In this study, we found significant low virologic failure (4.6%) and drug resistance (2.8%) among HIV-infected patients receiving first-line ART with the median duration of treatment of about 20-month in Guangxi, China. Also, virologic suppression increased over time. A recent systematic review and meta-analysis showed that the pooled prevalence of HIVDR was 11.1% (95% CI, 7.49–16.14%), which increased to 22.92% at 61–72 months (95% CI, 9.45–45.86%) among Chinese HIV-infected patients receiving first-line ART in China (13). Defining 1000 copies/ml as the same threshold of virologic failure, a systematic review conducted in Sub-Saharan Africa concluded that the rate of virologic suppression were 76 and 67% with the median duration of ART at 12- and 24-month, respectively (24). Based on another systematic review conducted in resource-limited settings in 2013, HIVDR at 12–23 months was 11.1% (25). With prolonged treatment, the rate of virologic failure and drug resistance increased over time (26, 27). In the 2012 WHO drug resistance surveillance guideline, viral load suppression after 12 months of ART can be graded as poor (<70%), fair (70–85%), or excellent (>85%) (28). The prevalence of virologic failure and drug resistance in our study is comparable to the virologic treatment responses seen in other developed and industrialized countries (4). What is more, of the 143 patients who were infected by drug injection, only 10 (7.0%) experienced virologic failure, lower than other provinces in China and this result consist with previous study in China (19). Compared to those results, we can conclude that successful outcomes of ART can be achieved in resource-limited settings.

There are several reasons to explain low virologic failure and drug resistance among HIV-infected patients receiving first-line ART in Guangxi. First, there are three levels of AIDS hospitals providing ART for HIV patients, including provincial, prefecture, and county hospitals. One provincial hospital provides technical supports and training to each prefecture hospital, which then provides technical assistance to each county hospital. Second, although HIV patients receive treatment in hospitals at or above the county level, health staff at village clinics or township hospitals are responsible for community care and outreach, such as home visits or telephone calls for reminding of ART adherence, and making an appointment if patients did not see doctors at AIDS hospitals for ART management every 3-month follow-up. Third, all doctors and health staff for ART and care management receive comprehensive HIV/AIDS medical training. Fourth, initial ART regimen with 3TC can increase virologic suppression. Our previous studies found HIV patients who received ART at village clinics, township hospitals, or local CDCs had poor virologic outcomes, whereas treatment counseling and instruction through telephone and 3TC-based regiments were significantly associated with HIV RNA suppression (16, 29, 30).

Despite successful HIV virologic suppression in this study, patients with missed doses in the past month and initial ART regiments without 3TC were found to be at increased risk for poor virologic response. This indicates that measures should be continuously taken to train local medical staff, and educate patients and enhance adherence monitoring. Due to the poor outcome of patients that received DDI-based treatment, the NFATP’s first-line antiretroviral regimens have been changed to exclude the use of didanosine (DDI) and to include the use of lamivudine (3TC) for all new patients beginning treatment since 2008 (7).

The prevalence of HIVDR among patients who experienced virologic failure was 62.9% (22/35). Of 22 patients with HIVDR, the proportions of NNRTI mutations, NRTI mutations, and PI mutations were 54.5, 90.0, and 4.5%, respectively. In addition, 10 (45.5%) were identified to be resistant to multi-drugs. M184I/V was the most common NRTI mutations and other studies in China showed similar results (16, 29). One PI mutation was identified in this study, because that PI was not scaled-up at that period.

The limitations can be summed up as follows. First, these results may not represent all patients in Guangxi because of the exclusion criteria and limited geographic distribution of the surveillance sites. Also, the surveys did not include all patients who have met the inclusion criteria in Guangxi, so when we draw the conclusion we should be cautious. Second, considering diverse HIV epidemic conditions in different districts in Guangxi, the difference among those districts should be considered.

In summary, this study in Guangxi demonstrates that HIV-infected patients receiving first-line treatment achieve an excellent treatment outcome and we can summarize those factors associated with virologic suppression as increasing the proper use of 3TC-based regimens and strengthening the importance of adhering to treatment.

## Conclusion

In this study, we found significant low virologic failure and drug resistance among HIV-infected patients receiving first-line ART with the median duration of treatment of about 20-month in Guangxi, China. Our study demonstrated that HIV-infected patients receiving first-line treatment can achieve an excellent treatment outcome in hospitals at or above the county level for free ART in Guangxi, and we can conclude that the increasing the proper use of 3TC-based regimens and the strengthening the importance of adhering to treatment are associated with virologic suppression.

## Author Contributions

JY and SL wrote the main manuscript text and FL, ZT, YS, and WL prepared Tables 1–3. WK, XL, LL, and YR collected the data. HX and YS designed the study. All authors read and approved the final manuscript.

## Conflict of Interest Statement

The authors declare that the research was conducted in the absence of any commercial or financial relationships that could be construed as a potential conflict of interest.
